# *Akkermansia muciniphila* and GLP-1-Based Therapies: Bidirectional Interactions and Implications for Type 2 Diabetes and MASLD/MASH

**DOI:** 10.3390/biomedicines14061235

**Published:** 2026-05-29

**Authors:** Boris Dinkov

**Affiliations:** 1 Department of Pharmacology and Toxicology, Medical University—Pleven, 5800 Pleven, Bulgaria; boris.dinkov@mu-pleven.bg; 2 Working Group “Scientific Guidance and Expert Support for the Implementation of Pharmacogenomics in Clinical Practice”, Project BG-RRP-2.004-0003, Medical University—Pleven, 5800 Pleven, Bulgaria; 3 Clinic of Endocrinology and Metabolic Diseases, University Hospital “Dr. Georgi Stranski”—Pleven, 5800 Pleven, Bulgaria

**Keywords:** *Akkermansia muciniphila*, GLP-1-based therapies, MASLD, MASH, gut microbiome, gut-liver axis, hepatoprotection, type 2 diabetes mellitus, microbiome remodeling

## Abstract

The global burden of type 2 diabetes mellitus (T2DM) and metabolic dysfunction-associated steatotic liver disease (MASLD) continues to rise at an alarming pace, with substantial pathophysiological overlap driven by insulin resistance, visceral obesity, and chronic low-grade inflammation. MASLD may progress to metabolic dysfunction-associated steatohepatitis (MASH), with increased risk of cirrhosis and hepatocellular carcinoma. Glucagon-like peptide 1 (GLP-1)-based therapies have transformed the management of T2DM and obesity. They exert pleiotropic effects whose basis remains incompletely understood. Concurrently, *Akkermansia muciniphila* has emerged as a keystone gut microbiota species with demonstrated hepatoprotective potential in preclinical models of MASLD/MASH. This narrative review positions *A. muciniphila* simultaneously as a target of GLP-1-mediated microbiome remodeling and as an independent modulator of hepatoprotection in MASLD/MASH. A structured search of PubMed, Scopus, and Web of Science (last searched: 12 April 2026) was conducted using terms related to *Akkermansia muciniphila*, GLP-1 receptor agonists, MASLD/MASH and T2DM. A total of 174 records were identified. Of these, 148 were excluded due to duplication or non-relevant study design. 26 studies (23 preclinical, 3 clinical) were included in the synthesis, directly addressing *A. muciniphila*. Preclinical evidence demonstrates that liraglutide, semaglutide, exenatide, and tirzepatide increase *A. muciniphila* abundance, while *A. muciniphila* in turn enhances endogenous GLP-1 secretion via the P9/ICAM-2 axis, forming a hypothetical positive feedback loop. A working mechanistic model integrating these bidirectional interactions is proposed, alongside a discussion of current limitations and future research priorities, including microbiome-guided clinical trials in MASLD/MASH populations.

## 1. Introduction

The global burden of type 2 diabetes mellitus (T2DM) and metabolic dysfunction-associated steatotic liver disease (MASLD) continues to rise at an alarming pace. Current estimates indicate that approximately 589 million people worldwide are living with diabetes, with T2DM accounting for over 90% of cases [1]. MASLD affects more than 60% of individuals with T2DM [2]. The metabolic and pathophysiological overlap between these two conditions is substantial. Insulin resistance, visceral obesity, and chronic low-grade inflammation represent the principal pathogenetic drivers [3]. MASLD may progress to metabolic dysfunction-associated steatohepatitis (MASH), characterized by hepatocellular injury, lobular inflammation, and fibrosis, which significantly increases the risk of cirrhosis and hepatocellular carcinoma [4]. The term MASLD replaced the earlier nomenclature of non-alcoholic fatty liver disease (NAFLD) in 2023 through a multidisciplinary consensus process [5], and both terms are used interchangeably in the older literature. NAFLD/NASH is retained in the present review when referring to studies published prior to this reclassification.

GLP-1-based therapies have substantially transformed the management of T2DM. Their use achieves effective glycemic control and body weight reduction and confers nephroprotective, hepatoprotective, and anti-inflammatory effects [6]. Recent clinical data with semaglutide 2.4 mg in patients with MASH (ESSENCE trial, 2025) confirmed histological improvement of steatohepatitis [7]. However, the mechanisms underlying these beneficial pleiotropic effects likely extend beyond direct receptor activation. A growing body of preclinical and clinical evidence indicates that GLP-1-based therapies exert significant modulatory effects on the composition and function of the gut microbiome, which may indirectly contribute to their hepatoprotective properties.

The gut-liver axis has emerged as an important concept in the pathophysiology of metabolic diseases, linking pathologically altered gut microbiota (dysbiosis) to hepatic inflammation, lipid dysregulation, and fibrogenesis [8]. Certain bacterial taxa exert a disproportionate influence on metabolic homeostasis relative to their abundance, so-called keystone species. Among the microorganisms colonizing the human intestinal mucosa, *Akkermansia muciniphila* has attracted considerable scientific interest owing to its pivotal role in maintaining the mucus layer and intestinal barrier integrity [9]. Available evidence indicates that its abundance is reduced in experimental animals with T2DM, obesity, and MASLD, and the same pattern has been observed in humans [10,11,12]. In experimental models, restoration of *A. muciniphila* abundance, whether through dietary intervention, pharmacotherapy, or direct supplementation, is associated with improved intestinal barrier integrity, reduced systemic endotoxemia, and attenuated hepatic inflammatory responses [13,14,15,16].

Available evidence suggests a plausible bidirectional nature of this relationship. GLP-1-based therapy is associated with increased *A. muciniphila* abundance in preclinical models, which may in turn enhance endogenous GLP-1 secretion [17,18]. This hypothesis, forming a potential positive feedback loop of therapeutic relevance, awaits direct clinical validation.

Despite accumulating data, existing publications address this topic only partially, focusing either on the bidirectional relationship between GLP-1 receptor agonists (GLP-1 RAs) and the gut microbiome in a broad metabolic context [19,20,21], or on the role of *A. muciniphila* as a next-generation probiotic in obesity and T2DM [22], or on its significance in MASLD/MASH independently of GLP-1-based therapies [13]. An integrated synthesis that simultaneously positions *A. muciniphila* as both a target of GLP-1-based therapy-mediated microbiome remodeling and an independent modulator of hepatoprotection in MASLD/MASH in the setting of T2DM and obesity is currently lacking. The present narrative review addresses this gap by integrating these perspectives within a unified mechanistic framework, alongside a discussion of clinical implications for microbiome-guided therapeutic strategies in MASLD/MASH.

*A. muciniphila* is examined as a keystone species—an organism exerting a disproportionately large influence on the gut microbiome relative to its abundance, within a hypothetical bidirectional GLP-1/microbiome axis. The available preclinical and clinical evidence regarding its role in GLP-1-based therapy-induced hepatoprotection in MASLD/MASH is summarized. Several factors justify the prioritization of *A. muciniphila* in the present review. It is a major gut commensal utilizing mucin as its primary substrate, occupying a unique mucosal niche with direct access to the intestinal epithelium. GLP-1-based therapies have been shown to specifically increase its abundance across multiple preclinical models. Pasteurized *A. muciniphila* has received regulatory authorization as a Novel Food in the European Union, representing a step toward translatability, achieved by only a few other next-generation gut-microbiota-derived microorganisms to date [23]. 

It should be emphasized that *A. muciniphila* represents only one of many microbial taxa involved in the regulation of the gut-liver axis and metabolic homeostasis. Several other bacteria, including *Faecalibacterium prausnitzii*, *Bifidobacterium* spp., *Lactobacillus* spp., and *Ruminococcus* spp., demonstrate hepatoprotective and anti-inflammatory effects through partially overlapping, yet also distinct, mechanisms [24]. Functional interactions within the gut microbiome indicate that changes in *A. muciniphila* abundance do not occur in isolation, but rather in the context of broader microbiome remodeling. The present review focuses specifically on *A. muciniphila* by virtue of its unique position within the mucus layer and the growing evidence for a bidirectional relationship with GLP-1-based therapies, without claiming an exclusive role in the microbiome-mediated regulation of hepatic pathology.

## 2. Materials and Methods

A structured literature search was conducted on 12 April 2026 across the PubMed, Scopus, and Web of Science databases. The following search terms and their combinations were used: “*Akkermansia muciniphila*”, “GLP-1 receptor agonists”, “MASLD”, “MASH”, “NAFLD”, “gut microbiome”, “gut-liver axis”, “type 2 diabetes mellitus”, “hepatoprotection”, “intestinal permeability”, “Amuc_1100”. Given that *A. muciniphila* gained metabolic relevance after 2013, articles published between 2013 and 2026 in the English language were considered. The initial search identified a total of 174 publications. Following removal of duplicates and screening of titles and abstracts based on relevance to the primary topic of the review, 26 publications (23 preclinical and 3 clinical) were included as directly relevant to the central research question and were prioritized in the synthesis. Additional references were included to support mechanistic context and background. Inclusion criteria encompassed original research articles addressing *Akkermansia muciniphila* in the context of T2DM, MASLD/MASH, or GLP-1-based therapy. Publications in languages other than English, those without access to full text, letters to the editor, commentaries, and case reports were excluded. Clinical studies in humans were prioritized where available. Preclinical and in vitro data were included to provide detailed description of mechanistic pathways and to support biological plausibility. Given the heterogeneity of the available data, a narrative review format was selected and no formal risk of bias assessment was performed. Methodological quality was considered during evidence synthesis, with preference given to peer-reviewed studies with adequate sample sizes, appropriate control groups, and clearly defined interventions and outcomes. Preclinical findings from single studies without independent replication were interpreted with particular caution.

## 3. *Akkermansia muciniphila*: Biology and Metabolic Significance

### 3.1. Taxonomy and Core Biological Characteristics

*A. muciniphila* is a Gram-negative, anaerobic, non-spore-forming bacterium belonging to the phylum *Verrucomicrobiota*, class *Verrucomicrobiae*, first isolated and described by Derrien et al. in 2004 from human fecal samples [25]. Historically, it was considered the sole representative of the genus *Akkermansia* in the human microbiome; however, contemporary genomic studies have demonstrated considerable taxonomic diversity within the genus, including the identification of multiple candidate species in humans [26,27,28]. The majority of studies in which *A. muciniphila* was directly administered employed the type strain MucT (DSM 22959). Several preclinical studies did not report strain identity, representing an additional source of heterogeneity. Studies investigating GLP-1-mediated changes in *A. muciniphila* abundance assessed endogenous microbial populations rather than administered strains, and strain-level characterization in this context was not performed.

*A. muciniphila* preferentially colonizes the mucus layer of the large intestine, utilizing mucin as its primary carbon and nitrogen source. A notable paradox has been described: while the bacterium degrades mucin, it simultaneously stimulates compensatory production of new mucin by colonic goblet cells [29,30]. A specific mechanism has recently been identified. The outer membrane protein Amuc_0904 may directly induce goblet cell differentiation, thereby leading to enhanced mucin production [31].

This apparent paradox is mechanistically explained by the kinetics of mucin turnover. *A. muciniphila* degrades existing mucin glycoproteins as a carbon and nitrogen source, while simultaneously stimulating compensatory de novo mucin synthesis through multiple mechanisms. The outer membrane protein Amuc_0904 has been shown to promote goblet cell differentiation [31], thereby increasing the cellular capacity for mucin production. Additionally, degradation products of mucin glycoproteins may serve as signaling molecules that further stimulate mucin gene expression in goblet cells. The net result is maintained or enhanced mucus layer thickness, provided that *A. muciniphila* colonization density remains within physiological bounds.

These biological characteristics, together with the bioactive components described below, form the basis for the growing interest in *A. muciniphila* in conditions such as type 2 diabetes mellitus, obesity, and MASLD/MASH, in the context of its metabolic significance.

### 3.2. Key Bioactive Components of Akkermansia muciniphila

**Figure 1:**
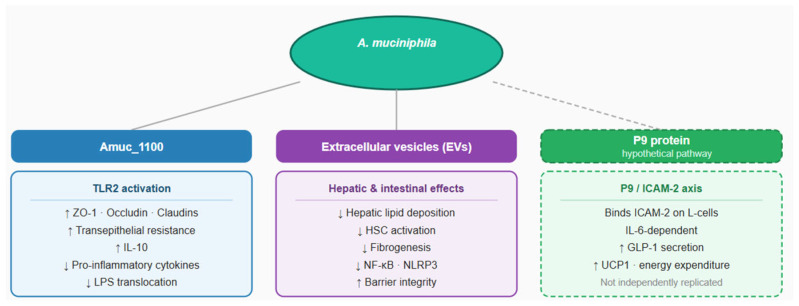
Key bioactive components of *Akkermansia muciniphila* and their principal mechanisms of action. Abbreviations: Amuc_1100—outer membrane protein of *Akkermansia muciniphila*; TLR2—Toll-like receptor 2; IL-10—interleukin-10; ZO-1—zonula occludens-1; P9—secreted protein P9 of *Akkermansia muciniphila*; IL-6–interleukin-6; ICAM-2—intercellular adhesion molecule-2; GLP-1—glucagon-like peptide-1; LPS—lipopolysaccharide; NF-κB—nuclear factor kappa B; NLRP3—NOD-like receptor thermal protein domain associated protein 3; HSC—hepatic stellate cells; UCP1 –uncoupling protein 1; ↑ indicates increase/upregulation, ↓ indicates decrease/downregulation.

The metabolic and hepatoprotective effects of *A. muciniphila* are mediated by several biologically active components, for which the greatest body of evidence is available for the proteins Amuc_1100 and P9, as well as extracellular vesicles (AmEVs) (Figure 1).

#### 3.2.1. The Role of Amuc_1100

Amuc_1100 is a well-characterized outer membrane protein of *A. muciniphila* with a unique structure. It is thermostable and retains its biological activity following pasteurization (30 min at 70 °C), the process by which the intact bacterium is treated to produce the pasteurized form of *A. muciniphila* [32].

In vitro studies in cell cultures and murine models have demonstrated that Amuc_1100 enhances intestinal barrier function by reinforcing tight junctions, upregulating the expression of occludin, claudin, and zonula occludens-1 (ZO-1) [33]. A study by Neurath et al. (2025) further confirmed this effect, identifying TLR2-mediated interaction as the underlying mechanism [34].

In vitro studies have established that Amuc_1100 predominantly activates TLR2, with evidence of additional interaction with TLR4, leading to upregulation of the anti-inflammatory cytokine IL-10 [35]. A recent study demonstrated that pre-treatment with Amuc_1100 significantly inhibited the expression of pro-inflammatory cytokines (TNF-α, IL-1β, IFN-γ, and IL-6) through suppression of the NF-κB signaling pathway in a murine model of acute pancreatitis [36]. Amuc_1100 may therefore participate in immunomodulatory interactions, achieving indirect anti-inflammatory effects.

The metabolic interactions of Amuc_1100 have been investigated in preclinical studies conducted in vitro (3T3-L1 preadipocyte cell lines) and in vivo in experimental animals [37]. In a murine study by Zheng et al. (2023), Amuc_1100 stimulated lipolysis and adipocyte browning through activation of the AC3/PKA/HSL signaling pathway, and additionally upregulated uncoupling protein 1 (UCP1) expression in brown adipose tissue, resulting in enhanced thermogenesis [37].

Amuc_1100 has also demonstrated antioxidant effects. In a study by Song et al. (2023), Amuc_1100 reduced malondialdehyde (MDA) and hydrogen peroxide levels, attenuating oxidative stress in *Salmonella typhimurium*-challenged mice through activation of the TLR2/NF-κB and Nrf2 signaling pathways [38]. Nrf2 is a central regulator of redox homeostasis in MASLD, where oxidative stress represents a key pathogenetic mechanism [39]. Nrf2 activation is associated with reductions in steatosis, lipid peroxidation, and hepatic inflammation [40]. The direct effect of Amuc_1100 on Nrf2 in MASLD requires independent validation in future studies. Translatability of the antioxidant effect of Amuc_1100 to metabolic disorders may be hypothesized, though remains theoretical at present.

All human studies have been conducted using the pasteurized form of *A. muciniphila*, which contains Amuc_1100 [41]. Despite available preclinical evidence, the independent effects of Amuc_1100 have not yet been validated in clinical trials, and further investigation in this direction is warranted.

#### 3.2.2. The P9 Protein

The P9 protein is a further component secreted by *A. muciniphila* with potential metabolic effects, albeit currently supported by preliminary, non-replicated preclinical evidence. First isolated by Yoon et al. (2021), P9 has been investigated in in vitro and in vivo preclinical studies [18]. In cell cultures of enteroendocrine L-cells, purified P9 protein can independently induce secretion of glucagon-like peptide-1 (GLP-1) [18]. The secreted endogenous GLP-1 mediates the so-called incretin effect, responsible for glucose-dependent insulin secretion from pancreatic beta cells [42]. P9 protein of *A. muciniphila* may therefore indirectly support improvement of glucose homeostasis. P9 has been shown to interact with intercellular adhesion molecule-2 (ICAM-2) on the surface of enteroendocrine L-cells [18]. This process is dependent on interleukin-6 (IL-6). In IL-6-deficient mice, ICAM-2 expression is reduced, which attenuates the effect of P9 on GLP-1 secretion and consequently diminishes its contribution to glucose homeostasis [18]. These findings confirm that IL-6-dependent expression of ICAM-2 is required for activation of the P9/ICAM-2 signaling axis. Additionally, P9 enhances thermogenesis and energy expenditure through upregulation of uncoupling protein 1 (UCP1) expression in brown adipose tissue [18]. It should be noted that the available data on P9 originate from a single research group and have not yet been independently replicated, necessitating cautious interpretation [18]. These observations position P9 as a potential candidate for the development of oral, targeted therapies against obesity and type 2 diabetes mellitus, albeit currently at a preclinical stage.

#### 3.2.3. Extracellular Vesicles

The extracellular vesicles (EVs) of *A. muciniphila* are spherical, bilayered lipid structures shed from its outer membrane, serving as key mediators of communication between the bacterium and the host [43]. EVs are composed of lipids (phospholipids, glycolipids), nucleic acids (DNA and RNA fragments), multiple proteins, enzymes and metabolites [43]. Under in vitro conditions, *A. muciniphila* EVs have been shown to activate the MAPK signaling pathway, thereby improving intestinal epithelial barrier integrity and exerting anti-inflammatory effects [43]. The putative hepatoprotective mechanisms involve EV interactions with TLR2 and TLR4 receptors and modulation of PPARα, PPARβ/δ, and PPARγ (peroxisome proliferator-activated receptors) expression [44].

The clinical translation of bacterially derived EVs, including those of *A. muciniphila*, faces substantial challenges common to the broader EV field. Inconsistent yields and vesicle heterogeneity remain unresolved, and standardized protocols for isolation, characterization, and dosing are currently lacking [45,46]. Targeted delivery is further constrained by rapid systemic clearance and predominant hepatosplenic biodistribution [47]. To date, no published clinical studies in humans have directly investigated the effects of isolated AmEVs on these signaling pathways.

EVs represent a promising, yet still insufficiently characterized effector system of *A. muciniphila* in humans, whose hepatoprotective potential requires direct clinical validation.

## 4. Abundance of *A. muciniphila* in Metabolically Healthy Individuals and Those with Metabolic Diseases

In healthy individuals, *Akkermansia muciniphila* constitutes approximately 1–4% of the total gut microbiota and is regarded as a marker of metabolic health. Colonization levels reach approximately 10^8^ cells/g of fecal matter in adults [48,49].

Multiple studies confirm that *A. muciniphila* abundance declines significantly with advancing age. A reduction in *A. muciniphila* and a concomitant increase in other bacteria, such as *Alistipes*, has been observed in elderly humans and experimental animals [50,51,52]. This decline is associated with impaired mucosal barrier function, reduced mucus layer thickness, systemic inflammation, and an increased prevalence of age-related metabolic disorders [53].

*A. muciniphila* abundance is reduced in metabolic diseases. Studies report an inverse correlation between *A. muciniphila* abundance and T2DM and obesity, with reduced levels observed in obese diabetic mice and humans [12,41]. Multiple studies demonstrate reduced abundance in non-alcoholic fatty liver disease and metabolic syndrome [41,54]. Greater *A. muciniphila* abundance is associated with lower body weight, lower HOMA-IR, and improved insulin sensitivity [41]. With regard to the lipid profile, higher *A. muciniphila* abundance is inversely correlated with plasma triglyceride levels, a relationship observed in both experimental models and clinical cohorts [11,55].

Despite these robust associations, causality has not been definitively established. Interventional studies in humans have yielded mixed results. A study by Zhang et al. (2025) [56] demonstrated that the efficacy of supplementation depends on baseline *A. muciniphila* levels. Patients with low baseline abundance showed significant improvements, whereas those with high baseline levels exhibited no significant effect [56]. The accumulated evidence indicates that this relationship is complex and likely modulated by the individual’s baseline microbial profile.

## 5. Bidirectional Interactions Between GLP-1-Based Therapies and *A. muciniphila* in MASLD/MASH: Preclinical and Clinical Evidence

### 5.1. Effects of A. muciniphila on Hepatic Homeostasis in MASLD/MASH—Preclinical Evidence

The accumulated preclinical evidence positions *A. muciniphila* as a modulator of hepatic homeostasis in MASLD/MASH. The key biological foundations and signaling pathways have been discussed in Section 3. The landmark studies of Everard et al. (2013) [12] and Plovier et al. (2017) [32] established *A. muciniphila* as a metabolically active microorganism with hepatoprotective potential in murine models of obesity, insulin resistance, and MASLD/MASH. A summary of the principal preclinical studies is presented in Table 1.

Of particular significance is the study by Plovier et al. (2017) [32]. The authors demonstrated that the pasteurized form is superior to the live bacterium in its ability to promote fat mass reduction, improve insulin sensitivity, and ameliorate lipid abnormalities. These findings challenged the prevailing paradigm of obligatory microbial viability for probiotic efficacy. The identification of Amuc_1100 as a TLR2-mediated effector remains central to subsequent mechanistic investigations.

Subsequent preclinical studies have demonstrated the hepatoprotective potential of *A. muciniphila* across various models of MASLD and MASH, mediated through several principal mechanisms, including modulation of lipid metabolism, modulation of bile acid signaling through activation of the Farnesoid X receptor—Fibroblast Growth Factor 15 (FXR-FGF15) pathway [60], and enhanced mitochondrial oxidation via L-aspartate signaling [58]. A systematic review by Asghari et al. (2025) [13] encompassing 13 preclinical studies confirmed that *A. muciniphila* reduces hepatic steatosis and serum lipid levels in MASLD/MASH models. Key mechanisms include decreased hepatic triglyceride synthesis, improved insulin sensitivity, and anti-inflammatory effects [13].

Suppression of hepatic inflammation has been demonstrated through inhibition of NLRP3 inflammasome activity and attenuation of TLR4/NF-κB signaling [14]. NLRP3 is a mediator of IL-1β-dependent hepatic inflammation and fibrogenesis. A study by Han et al. (2023) reported normalization of pathologically hyperactivated hepatic TLR2 signaling and a shift in macrophage polarization from the M1 to the M2 phenotype [59]. Raftar et al. (2022) [15] identified antifibrotic activity of *A. muciniphila* in a HFD/CCl4 murine model—a combined approach using high-fat diet and carbon tetrachloride to induce MASH with accelerated fibrosis. EVs of *A. muciniphila* demonstrated particularly potent inhibition of hepatic stellate cell activation, with isolated EV administration showing more pronounced modulation of TLR and PPAR gene expression in the liver compared to both the live and pasteurized forms [15]. In the recently published MASLD model of González-Robles et al. (2026), *A. muciniphila* was administered alone and in combination with melatonin, demonstrating a hepatoprotective effect that was more pronounced when combined with melatonin [61].

Kwak et al. (2026) identified *Romboutsia hominis* as a novel mediator of MASH progression, while *A. muciniphila* and its EVs were identified as hepatoprotective factors [62]. The authors additionally proposed a mechanism by which EVs may reduce hepatic lipid accumulation through downregulation of lipid biosynthesis-related genes.

The preclinical evidence base demonstrates hepatoprotective effects of *A. muciniphila* in MASLD/MASH models, mediated through mechanisms that enhance intestinal barrier function, improve bile acid metabolism, and modulate immune responses and fibrogenesis. These observations, however, remain largely confined to experimental models.

A key question is whether GLP-1-based therapies, already established in clinical practice for T2DM and obesity, can increase *A. muciniphila* abundance in vivo and thereby augment their hepatoprotective potential as part of their pleiotropic mechanism of action.

### 5.2. Effects of GLP-1-Based Therapies on A. muciniphila Abundance

The evidence reviewed in this section derives predominantly from animal models. To date, no controlled human trial has demonstrated a specific effect of GLP-1-based therapies on *A. muciniphila* abundance, and the findings described below should be interpreted within this preclinical context.

In addition to their direct metabolic effects, GLP-1-based therapies exert modulatory activity on the gut microbiome. Among the taxa potentially influenced by these therapies, *A. muciniphila* is among those identified in preclinical models. A summary of the principal preclinical studies is presented in Table 2.

In murine models of diet-induced obesity and type 2 diabetes mellitus, liraglutide increases the relative abundance of *A. muciniphila*, with concomitant improvements in glycemic control and reductions in body weight [64,66]. Semaglutide demonstrates a similar profile, with restoration of reduced *A. muciniphila* abundance observed in two independent preclinical models of HFD-induced obesity [68,69]. In a study by Chen et al. (2023), exenatide intervention in diabetic mice showed a significant increase in *A. muciniphila* alongside a simultaneous reduction in pathogenic bacteria [67].

Of growing interest are data on tirzepatide—a dual agonist of GLP-1 and GIP receptors. In two independent preclinical studies, tirzepatide significantly increased *A. muciniphila* abundance. Hu et al. (2025) demonstrated that in db/db mice with MASLD, tirzepatide achieved a more pronounced increase in *A. muciniphila* and more effective reduction in hepatic steatosis compared to semaglutide [71]. Wang et al. (2025) confirmed these observations in an HFD model, identifying a negative correlation between *A. muciniphila* abundance and degree of adiposity following tirzepatide intervention [73]. These data suggest that the additional GIP receptor agonism may augment microbiome remodeling toward increased *A. muciniphila* abundance, although direct comparison with monoagonists requires further validation.

It is important to note that not all preclinical studies document a specific effect on *A. muciniphila*. In some, broader changes in microbiome composition were observed without specific reporting of this taxon [63,65,70,72], underscoring the need for standardized methodological approaches in microbiome data analysis.

In the context of this bidirectional axis, it should be recalled that *A. muciniphila* is not solely a target of GLP-1-based therapy-mediated microbiome remodeling. It may itself amplify endogenous GLP-1 secretion through the secreted protein P9, acting on enteroendocrine L-cells via the ICAM-2 receptor [18]. At physiological IL-6 concentrations, classical signaling through the membrane-bound IL-6 receptor (IL-6R) promotes insulin secretion, lipolysis, and fatty acid oxidation [76]. Chronically elevated IL-6, as observed in metabolic diseases including MASLD/MASH, preferentially activates trans-signaling through the soluble IL-6 receptor (sIL-6R), inducing pro-inflammatory effects, enhanced insulin resistance, and hepatic fibrogenesis [76]. Whether the chronic IL-6 elevation characteristic of advanced MASLD amplifies or attenuates P9-mediated GLP-1 secretion remains to be established in future studies.

Of particular significance is the preclinical study by Gao et al. (2026), which demonstrated that combined administration of semaglutide and *A. muciniphila* strain Akk11 in db/db mice with MASLD produced a synergistic effect on metabolic parameters, achieved through gut microbiota remodeling [75]. Akk11 is a specific strain isolated from fecal samples of healthy infants [77]. This constitutes preclinical evidence supporting the combined use of GLP-1-based therapy and *A. muciniphila*.

This preclinical-clinical discrepancy is further illustrated by the available human data. In a randomized, double-blind, placebo-controlled trial by Smits et al. (2021) enrolling 51 patients with T2DM, liraglutide 1.8 mg demonstrated no effect on alpha or beta diversity of the gut microbiota after 12 weeks of treatment [78]. The remaining clinical studies document broader changes in microbiome composition with various GLP-1 RAs, but do not specifically report *A. muciniphila* abundance [79,80]. The study by Klemets et al. (2026) offers an interesting perspective, with authors finding that baseline microbiota predicts therapeutic response to semaglutide and empagliflozin (an SGLT2 inhibitor), without, however, specifically investigating *A. muciniphila* [81].

The heterogeneity of clinical findings may be attributed to several factors. Existing studies differ substantially in design, duration, agent used, and microbiome analytical methodology. Changes in dietary behavior and reduced caloric intake associated with GLP-1-based therapy may independently influence microbiome composition, complicating the distinction between the direct effects of GLP-1-based agents and secondary effects resulting from improved glycemic control and body weight reduction [17]. Notably, none of the available clinical studies have been conducted in a MASLD/MASH population—precisely the population most likely to exhibit reduced baseline *A. muciniphila* abundance and therefore the highest potential for microbiome remodeling under GLP-1-based therapy.

The proposed mechanisms by which GLP-1-based therapies may increase *A. muciniphila* abundance encompass several complementary pathways. The most robustly supported is the effect on mucin secretion. GLP-1 receptors are expressed in Brunner’s glands, whose activation by GLP-1 agonists leads to increased mucin secretion and upregulated mucin 5b expression [82], thereby expanding the ecological niche of *A. muciniphila*, which utilizes mucin as its primary substrate. Additional indirect mechanisms include delayed gastric emptying [83], which modifies intestinal transit and pH in a direction potentially favorable for *A. muciniphila* colonization in the large intestine [84]. GLP-1 RA-induced changes in bile acid metabolism [19] may further modulate the gut microbiome. Reduction in chronic low-grade inflammation and improved intestinal mucosal integrity achieved with GLP-1-based therapy [85] may indirectly sustain the mucosal niche of *A. muciniphila*. The strength of preclinical and clinical evidence for each mechanistic domain is summarized in Table 3.

## 6. Role of *A. muciniphila* in the Hepatoprotective Effects of GLP-1-Based Therapies in MASLD/MASH

The available clinical evidence regarding the effects of *A. muciniphila* in humans is limited, yet conceptually significant. Dao et al. (2016) demonstrated that higher baseline *A. muciniphila* levels correlate with a more favorable metabolic response to caloric restriction [11], suggesting that baseline microbial status may predict therapeutic response. The study by Depommier et al. (2019) demonstrated that pasteurized *A. muciniphila* improves insulin sensitivity and reduces serum markers of hepatic dysfunction in humans, despite the limitations of the small cohort [41]. These findings raise an important hypothesis. Could GLP-1-induced increases in *A. muciniphila* abundance in MASLD patients, a population with reduced baseline levels, convert them from a low-responder to a high-responder phenotype? This hypothesis remains uninvestigated in clinical settings and represents a priority direction for future research.

The following working model integrates the available preclinical and clinical data. It is proposed as a conceptual framework and requires prospective clinical validation. GLP-1-based therapies exert hepatoprotective effects in MASLD/MASH through both direct and indirect mechanisms. Direct mechanisms encompass receptor-mediated effects, including enhanced insulin secretion from pancreatic β-cells, which improves glycemic control and reduces β-cell lipotoxicity. Regulation of food intake leads to reduced caloric consumption, body weight reduction, and decreased hepatic lipid accumulation. The indirect pathway operates through microbiome remodeling, with increased *A. muciniphila* abundance, which exerts independent hepatoprotective effects through improvement of intestinal barrier function, modulation of bile acid signaling, suppression of hepatic inflammation, and antifibrotic activity. Increased *A. muciniphila* abundance may further amplify endogenous GLP-1 secretion via the P9/ICAM-2 mechanism. This pathway is currently supported by preliminary, non-replicated preclinical evidence and requires independent validation.

**Figure 2:**
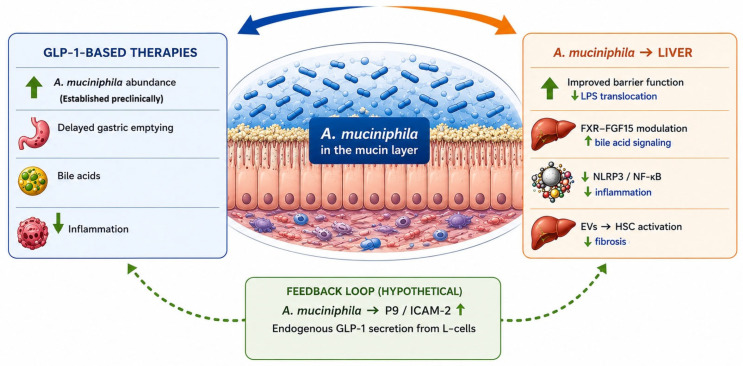
Bidirectional interactions between GLP-1-based therapies and *Akkermansia muciniphila* in the context of hepatoprotection in MASLD/MASH: proposed mechanistic model. Solid blue and orange arrows indicate established interactions supported by preclinical evidence. The dashed green arrow represents the hypothetical feedback loop (P9/ICAM-2 axis), which is based on preliminary, non-replicated preclinical evidence and has not been validated in human studies. Abbreviations: GLP-1—glucagon-like peptide-1; LPS—lipopolysaccharide; FXR—farnesoid X receptor; FGF15—fibroblast growth factor 15; NLRP3—NOD-like receptor thermal protein domain associated protein 3; NF-κB—nuclear factor kappa B; EVs—extracellular vesicles; HSC—hepatic stellate cells; ICAM-2—intercellular adhesion molecule-2; ↑ indicates increase/upregulation, ↓ indicates decrease/downregulation.

Through these complementary mechanisms, *A. muciniphila* is positioned as a putative integral component of the pleiotropic action of GLP-1-based therapies, pending direct clinical confirmation (Figure 2).

## 7. Discussion

The present review integrates preclinical and clinical evidence to examine *A. muciniphila* within a bidirectional GLP-1/microbiome axis in the context of MASLD/MASH and T2DM. The preclinical evidence base is substantial and mechanistically coherent. The translation of these observations to human clinical practice remains premature, and the available clinical evidence is considerably limited.

Several factors contribute to this preclinical-clinical discrepancy. The gut microbiome is characterized by substantial inter-individual variability. Key determinants include age, dietary patterns, geographic origin, and concomitant medication use. These include antibiotics, proton pump inhibitors, metformin, probiotics and prebiotics. The sequencing methodology employed, whether 16S rRNA sequencing or shotgun metagenomics, introduces additional analytical heterogeneity. This variability complicates both the standardization of microbiome endpoints and the interpretation of results across studies. *A. muciniphila* abundance has rarely been included as a pre-specified primary or secondary endpoint in clinical trials of GLP-1-based therapies, limiting the available evidence to post hoc or exploratory analyses.

The regulatory framework governing microbiome-based interventions remains underdeveloped, creating additional barriers to the design and approval of interventional studies. The absence of validated, non-invasive biomarkers for microbiome stratification and for the assessment of hepatic inflammation and fibrosis in MASLD/MASH further hampers the translation of preclinical findings into clinical trial design. These factors explain why the clinical evidence base remains insufficient despite the biological plausibility of the proposed model.

### 7.1. Mechanistic Integration

The working mechanistic model proposed in Section 6 integrates direct and indirect hepatoprotective pathways of GLP-1-based therapies, with *A. muciniphila* as a putative mediator of the latter. A critical and currently unresolved mechanistic question is whether the observed increases in *A. muciniphila* abundance under GLP-1-based therapy represent a direct pharmacological effect of GLP-1 receptor activation, or whether they are secondary consequences of weight loss, improved glycemic control, reduced caloric intake, or dietary changes that accompany treatment. Several lines of evidence support a plausible direct mechanism: GLP-1 receptors are expressed in Brunner’s glands, whose activation leads to increased mucin secretion and expanded ecological niche for *A. muciniphila*. In addition, the effect has been observed across multiple structurally distinct agents, including liraglutide, semaglutide, exenatide, and tirzepatide. Weight loss per se is an established independent modulator of gut microbiome composition, and reduced caloric intake during GLP-1 therapy may independently favor mucin-degrading bacteria. Dissociating the direct drug effect from these confounders requires controlled human studies assessing microbiome composition in parallel with body weight, glycemic parameters, and dietary intake. Such a study design has not yet been implemented in a MASLD/MASH population with *A. muciniphila* as a pre-specified endpoint. The P9/ICAM-2 signaling axis warrants particular caution in interpretation. This pathway remains subject to the replication caveat described in Section 3.2.2.

As detailed in Section 5.2, the dual signaling profile of IL-6, classical versus trans-signaling, is directly relevant to the P9/ICAM-2 axis. Whether the chronic IL-6 elevation characteristic of advanced MASLD attenuates or fundamentally modifies P9-mediated GLP-1 secretion remains unresolved. These uncertainties necessitate cautious interpretation of the proposed feedback loop until independent replication and human validation are available.

### 7.2. Clinical Implications and Future Therapeutic Strategies

The available evidence, while predominantly preclinical, provides a conceptual framework for several clinically relevant therapeutic strategies that merit prospective investigation. Future randomized studies in selected human populations, with *A. muciniphila* abundance as a pre-specified endpoint and stratification by baseline microbiome profile, are required for direct validation of the proposed model. Of particular interest is the combined administration of GLP-1-based therapies with pasteurized *A. muciniphila*. This approach is supported by the preclinical synergistic data of Gao et al. (2026) [75]. A MASLD/MASH population represents the most appropriate target for such trials, given the consistently documented reduction in baseline *A. muciniphila* abundance in this group and the established histological efficacy of semaglutide in this indication.

Determination of the baseline microbiome profile as a predictor of therapeutic response represents a promising clinical concept. Metagenomic sequencing, via shotgun sequencing or 16S rRNA amplicon sequencing, provides the methodological basis for quantitative assessment of *A. muciniphila* prior to treatment initiation. The baseline-dependent pattern documented by Zhang et al. (2025) [56] raises the hypothesis that GLP-1-induced microbiome remodeling may exhibit analogous response kinetics. Specifically, patients with low baseline *A. muciniphila* abundance may demonstrate greater microbiome remodeling and, consequently, more effective hepatoprotection. Implementation of this concept would require standardization of analytical methods, as well as integration of microbiome data with validated non-invasive biomarkers for the assessment of hepatic inflammation and fibrosis.

Dietary and postbiotic strategies offer a complementary approach to increasing *A. muciniphila* abundance. Several prebiotics have demonstrated the capacity to increase its abundance, including inulin, galacto-oligosaccharides, polyphenols, and arabinoxylans [86,87]. The synergistic effect between dietary fiber and polyphenols simultaneously improves microbial diversity and short-chain fatty acid production [88]. With regard to postbiotics, pasteurized *A. muciniphila* provides a regulatory-validated approach to delivering bioactive components [32]. It should be noted that the effect of prebiotics on *A. muciniphila* is not universal and depends on the individual’s baseline microbiome profile [89], underscoring the need for a personalized approach.

The safety and regulatory profile of *A. muciniphila* represents an important consideration for its potential clinical application. In the preclinical study by Plovier et al. (2017) [32], live *A. muciniphila* demonstrated a comparable safety profile to the pasteurized form, although its metabolic efficacy was less pronounced. In the study of Depommier et al. (2019) [41], both live and pasteurized *A. muciniphila* were evaluated in humans, with no serious adverse events reported in either group during a three-month supplementation period. Caution may be warranted in immunocompromised individuals, as the theoretical risk of bacterial translocation associated with live microbial preparations cannot be entirely excluded in the setting of impaired mucosal barrier integrity or immune dysfunction. From a regulatory perspective, pasteurized *A. muciniphila* received Novel Food authorization in the European Union following a positive safety assessment by the EFSA in 2021 [23]. This represents one of the earliest regulatory approvals involving a next-generation probiotic-based product. This authorization applies specifically to pasteurized preparations, whereas live formulations, extracellular vesicle-derived products, and other postbiotic approaches remain at preclinical or early clinical development stages. Important methodological challenges remain unresolved, including standardization of manufacturing processes, dosing strategies, viability assessment, and long-term safety evaluation. Further clarification of these aspects is required before broader clinical implementation can be considered.

### 7.3. Limitations

The present review is subject to several important limitations that constrain the strength of its conclusions. The preclinical data are derived predominantly from HFD and db/db murine models, which do not adequately reproduce the complex pathophysiology of metabolic disorders in humans. Translation of these findings is further complicated by fundamental differences in microbiome composition between rodents and humans, including differences in gut anatomy, diet, and colonization history that limit the direct applicability of murine microbiome data to human disease. Furthermore, strain-specific differences in metabolic or hepatoprotective effects between strains such as Akk11 and MucT cannot be excluded.

A causal relationship between GLP-1 RA-induced increases in *A. muciniphila* abundance and hepatoprotective effects has not been directly established. The observed microbiome changes may represent a secondary consequence of improved metabolic parameters, such as body weight reduction and improved glycemic control, rather than an independent pharmacological mechanism. In humans, a specific effect of GLP-1 RAs on *A. muciniphila* has not been documented in controlled trials, and clinical data on *A. muciniphila* in a MASLD/MASH population are entirely absent.

The focus on *A. muciniphila*, while justified by the growing evidence for its bidirectional relationship with GLP-1-based therapies, represents an inherent simplification of the gut microbiome’s complexity. Other hepatoprotective taxa, including *Faecalibacterium prausnitzii*, *Bifidobacterium* spp., and *Ruminococcus* spp., interact with *A. muciniphila* within a complex ecological network. Changes in its abundance do not occur in isolation from broader microbiome remodeling. These interactions are not captured comprehensively in the present review. The proposed positive feedback loop between *A. muciniphila* and endogenous GLP-1 secretion via the P9/ICAM-2 axis remains a hypothesis and requires direct clinical validation. No clinical implications should be drawn from it until independent replication in human studies is available.

## 8. Conclusions

The present review proposes an integrated model in which *A. muciniphila* functions as a keystone species within the bidirectional GLP-1/microbiome axis. Its role encompasses both a target of GLP-1-mediated microbiome remodeling and an independent modulator of hepatic homeostasis in MASLD/MASH in the setting of T2DM and obesity. Preclinical evidence supports the hepatoprotective potential of *A. muciniphila* in MASLD/MASH through multiple mechanisms. GLP-1-based therapies increase *A. muciniphila* abundance in animal models, though this effect has not been consistently demonstrated in controlled human trials. Preclinical data suggest potential therapeutic complementarity of their combined use, pending clinical validation. Clinical data, although limited, introduce the concept of baseline-dependent efficacy. This may explain the heterogeneity in therapeutic response and represent a conceptual basis for future microbiome-guided personalization of treatment in MASLD/MASH.

Validation of the proposed model requires clinical studies in a MASLD/MASH human population in which *A. muciniphila* abundance is a pre-specified endpoint. Direct evaluation of the combined use of GLP-1-based therapy with live or pasteurized *A. muciniphila* represents a priority research direction. Development of non-invasive biomarkers for microbiome stratification represents a further research priority. Integration of prebiotic and postbiotic approaches into therapeutic algorithms for MASLD/MASH warrants prospective evaluation. *A. muciniphila* represents a compelling, yet clinically insufficiently validated, therapeutic target at the intersection of the gut microbiome, the incretin system, and MASLD/MASH in T2DM. Its role in GLP-1-mediated hepatoprotection requires direct confirmation in prospective human studies before clinical translation can be considered.

## Figures and Tables

**Table 1:** Preclinical studies, evaluating *A. muciniphila* in animal models of obesity and MASLD.

Study	Model	Intervention (Dose, Duration)	Key Findings	Proposed Mechanisms
Everard et al. (2013) [12]	Ob/ob C57BL/6 mice with HFD	Live *A. muciniphila* (2 × 10^8^ CFU/daily) 4 weeks	Reduction in fat mass and adipose tissue inflammation markers; improved insulin sensitivity	Control of inflammation, intestinal barrier, and gut peptide secretion
Plovier et al. (2017) [32]	Mice on HFD and diabetes	Live (10^9^ bacterial cells/daily) or pasteurized *A. muciniphila* (2 × 10^8^ bacterial cells daily), 5 weeks	Pasteurized form superior to live bacteria in reducing fat mass and improving insulin sensitivity	Amuc_1100 improves intestinal barrier; participates in modulation of metabolomic profile
Kim et al. (2020) [57]	C57BL/6N mice, HFD	Live *A. muciniphila* (10^8^ CFU/mL) 10 weeks	Reduction in serum TGs, ALT; prevention of hepatic steatosis	Regulation of hepatic triglyceride synthesis;
Rao et al. (2021) [58]	C57BL/6 mice HFC diet	Live *A. muciniphila* (2 × 10^8^ CFU/daily) 8 weeks	Reduction in hepatic steatosis and inflammation	Enhanced lipid oxidation
Raftar et al. (2022) [15]	Mice HFD and CCl_4_	Live, pasteurized *A. muciniphila* (2 × 10^8^ CFU/daily) or EVs 4 weeks	Inhibition of hepatic inflammation.	Improved intestinal integrity; anti-inflammatory effects in liver and adipose tissue
Han et al. (2023) [59]	Mice with HFD-induced NASH	Live *A. muciniphila* (2 × 10^8^ CFU/daily, 8 weeks)	Prevention of hepatic inflammation;	Improved intestinal barrier; attenuated hepatic TLR2 hyperactivation;
Wu et al. (2023) [60]	C57BL/6 mice HFD	Live *A. muciniphila* (2 × 10^8^ CFU/daily, 8 weeks)	Reduction in body weight, hepatic steatosis, and liver injury; improved glucose tolerance.	Modulation of gut microbiome and bile acids; regulation of the FXR-FGF15 axis;
Qu et al. (2023) [14]	Mice HFD	Live and pasteurized *A. muciniphila* (2 × 10^8^ CFU/daily) + Amuc_1100 (100 μg/daily) 10 weeks	Reduction in body weight and serum ALT/AST; improved serum lipids	Action on the gut-liver axis; reduction in NLRP3 and TLR4/NF-κB expression
González-Robles et al. (2026) [61]	C57BL/6J mice on Western diet + fructose + CCl_4_ (MASLD-associated fibrosis)	Four groups: (1) no intervention; (2) melatonin alone; (3) *A. muciniphila* alone (2 × 10^8^ CFU/day); (4) melatonin + *A. muciniphila* combination; 4 weeks	Both interventions (alone and combined) showed hepatoprotective effects and partial restoration of gut microbiome	Modulation of hepatic and intestinal gene expression; synergistic effects of *A. muciniphila* + melatonin
Kwak et al. (2026) [62]	Mice MASH model	*A. muciniphila* and EVs	Reduction in hepatic lipid accumulation and inflammation;	Downregulation of lipid biosynthesis-related genes; modulation of TNF-α signaling

Abbreviations: HFD—high-fat diet; HFC—high-fat, high-cholesterol diet; CCl_4_—carbon tetrachloride; CFU—colony-forming units; EVs—extracellular vesicles; ALT—alanine aminotransferase; AST—aspartate aminotransferase; NASH—non-alcoholic steatohepatitis; MASLD—metabolic dysfunction-associated steatotic liver disease; MASH—metabolic dysfunction-associated steatohepatitis; TLR2—Toll-like receptor 2; TGs—triglycerides; NF-κB—nuclear factor kappa B; NLRP3—NOD-like receptor thermal protein domain associated protein 3; FXR—farnesoid X receptor; FGF15—fibroblast growth factor 15; TNF-α—tumor necrosis factor alpha; C57BL/6J/C57BL/6N—inbred mouse strains commonly used in metabolic research; ob/ob—leptin-deficient obese mouse model.

**Table 2:** Preclinical studies investigating the effect of GLP-1-based therapies on *A. muciniphila* abundance.

Author, Year	Model	Drug, Dose, Duration	Reported Outcome
Wang et al., 2016 [63]	C57BL/6J mice with diet-induced obesity	Liraglutide, dose not specified, 8 weeks	Modulation of gut microbiota with changes in weight-relevant phylotypes; *A. muciniphila* not specifically reported
Moreira et al., 2018 [64]	ob/ob mice and C57BL/6J mice with HFD	Liraglutide, 400 μg/kg/daily s.c., 8 weeks	Significant increase in *Akkermansia muciniphila* in the HFD group treated with liraglutide
Madsen et al., 2019 [65]	C57BL/6J mice with diet-induced obesity (DIO)	Liraglutide 0.2 mg/kg twice daily or GUB09-145 (dual GLP-1/GLP-2) 0.04 mg/kg twice daily, 4 weeks	Discrete changes in low-abundance species; no specific increase in *A. muciniphila* reported
Liu et al., 2020 [66]	db/db mice (NAFLD model)	Liraglutide, 200 μg/kg/daily s.c., 4 weeks	Significant increase in *Akkermansia* abundance
Chen et al., 2023 [67]	C57BL/6J diabetic mice (STZ-induced diabetes)	Exenatide, 24 nmol/kg/daily s.c., 8 weeks	Increase in *Akkermansia*; reduction in pathogenic bacteria (*Streptococcaceae*, *Erysipelotrichaceae*)
Duan et al., 2024 [68]	C57BL/6J mice high fat diet	Semaglutide, 30 μg/kg/daily s.c., 18 days	Restoration of HFD-reduced *Akkermansia* abundance; negative correlation with body weight and glucose
Feng et al., 2024 [69]	C57BL/6J obese mice (HFD)	Semaglutide, 30 μg/kg/daily s.c., 12 weeks	Increase in *Akkermansia* abundance
Mao et al., 2024 [70]	db/db mice (MASLD model)	Semaglutide, 10 nmol/kg twiceweekly s.c., 8 weeks	Changes in gut microbiota (*Alloprevotella*, *Alistipes*, *Ligilactobacillus*, *Lactobacillus*); *A. muciniphila* not specifically reported
Hu et al., 2025 [71]	db/db diabetic mice (MASLD model)	Tirzepatide 10 nmol/kg twice weekly s.c. vs. Semaglutide 10 nmol/kg twice weekly s.c., 8 weeks	Significant increase in *Akkermansia muciniphila*; tirzepatide more effective than semaglutide for hepatic steatosis and microbial modulation
Sun et al., 2025 [72]	C57BL/6J mice HFD	Semaglutide, 100 μg/kg/daily s.c., 12 weeks	Changes in gut microbiota composition; *A. muciniphila* not specifically reported
Wang et al., 2025 [73]	C57BL/6J mice HFD	Tirzepatide, 10 nmol/kg twice weekly s.c., 2 weeks	Restoration of HFD-reduced *Akkermansia* abundance; negative correlation with body weight, glucose, and adiposity
Zhang et al., 2025 [74]	KKay mice (prediabetes model)	Liraglutide, 200 μg/kg/daily i.p., 12 weeks	Changes in gut microbiota (reduction in *Ruminococcaceae*, *Anaerotruncus*); *A. muciniphila* not specifically reported
Gao et al., 2026 [75]	db/db mice (T2DM and MASLD)	Semaglutide 10 nmol/kg 2 twice weekly s.c. + *A. muciniphila* Akk11 10^9^ CFU/daily, 8 weeks	Synergistic effect: combination superior to monotherapy; microbiota remodeling and improved hepatic histology

Abbreviations: HFD—high-fat diet; DIO—diet-induced obesity; STZ—streptozotocin; s.c.—subcutaneous; i.p.—intraperitoneal; CFU—colony-forming units; GLP-1—glucagon-like peptide-1; NAFLD—non-alcoholic fatty liver disease; MASLD—metabolic dysfunction-associated steatotic liver disease; T2DM—type 2 diabetes mellitus; C57BL/6J—inbred mouse strain commonly used in metabolic research; ob/ob—leptin-deficient obese mouse model; db/db—leptin receptor-deficient mouse model of type 2 diabetes and obesity; KKAy—polygenic mouse model of type 2 diabetes and obesity with ectopic expression of the agouti gene.

**Table 3:** Comparison of preclinical and clinical evidence for key mechanisms.

Mechanism	Preclinical	Clinical
↑ *A. muciniphila* by GLP-1 RAs	Consistent	Absent
↓ Hepatic steatosis	Robust	Limited
↑ Barrier integrity	Established	Indirect
↓ Inflammation	Established	Absent
↓ Fibrosis (EVs)	Established	Absent
Bile acid modulation	Single study	Absent
P9/ICAM-2 → GLP-1	Single group	Absent
Pasteurized *A.muciniphila* supplementation	Established	Preliminary
Baseline-dependent efficacy	Not assessed	Available
Combined GLP-1 + *A.muciniphila*	Preclinical	Absent

↑ indicates increase/upregulation; ↓ indicates decrease/downregulation.

## Data Availability

No new data were created or analyzed in this study.

## Abbreviations

AC3	Adenylyl cyclase 3
ALT	Alanine aminotransferase
AmEVs	*Akkermansia muciniphila* extracellular vesicles
AST	Aspartate aminotransferase
BAT	Brown adipose tissue
CFU	Colony-forming units
DIO	Diet-induced obesity
EVs	Extracellular vesicles
FGF15	Fibroblast growth factor 15
FXR	Farnesoid X receptor
GIP	Glucose-dependent insulinotropic polypeptide
GLP-1	Glucagon-like peptide-1
GLP-1	RA Glucagon-like peptide-1 receptor agonist
HFC	High-fat, high-cholesterol diet
HFD	High-fat diet
HOMA-IR	Homeostatic model assessment of insulin resistance
HSC	Hepatic stellate cells
HSL	Hormone-sensitive lipase
ICAM-2	Intercellular adhesion molecule-2
IL	Interleukin
LPS	Lipopolysaccharide
MAPK	Mitogen-activated protein kinase
MASH	Metabolic dysfunction-associated steatohepatitis
MASLD	Metabolic dysfunction-associated steatotic liver disease
MDA	Malondialdehyde
NAFLD	Non-alcoholic fatty liver disease
NASH	Non-alcoholic steatohepatitis
NF-κB	Nuclear factor kappa B
NLRP3	NOD-like receptor thermal protein domain associated protein 3
Nrf2	Nuclear factor erythroid 2-related factor 2
PKA	Protein kinase A
PPAR	Peroxisome proliferator-activated receptor
SGLT2	Sodium-glucose cotransporter 2
sIL-6R	Soluble interleukin-6 receptor
STZ	Streptozotocin
T2DM	Type 2 diabetes mellitus
TLR2	Toll-like receptor 2
TLR4	Toll-like receptor 4
TNF-α	Tumor necrosis factor alpha
UCP1	Uncoupling protein 1
WAT	White adipose tissue
ZO-1	Zonula occludens-1
